# Development and validation of a machine learning model for predicting 3-year recurrence of adenomatous colorectal polyps after EMR: a multicenter study

**DOI:** 10.3389/fonc.2026.1936137

**Published:** 2026-09-09

**Authors:** Xiaoting Wu, Wenling Li, Dingmin Wang, Lu Liu, Yiheng Shi, Sujuan Fei

**Affiliations:** 1 Department of Gastroenterology, The Affiliated Hospital of Xuzhou Medical University, Xuzhou, China; 2 Medical College of Xuzhou Medical University, Xuzhou, China; 3 Department of Gastroenterology, The Third Affiliated Hospital of Xuzhou Medical University, Xuzhou, China; 4 Key Laboratory of Gastrointestinal Endoscopy, Xuzhou Medical University, Xuzhou, China

**Keywords:** adenoma, machine learning, online calculator, predictive model, recurrence

## Abstract

**Objective:**

This study aimed to identify factors associated with adenoma recurrence within three years after endoscopic mucosal resection (EMR) and to develop an individualized predictive model.

**Methods:**

Patients undergoing their first EMR for colorectal polyps at the Affiliated Hospital of Xuzhou Medical University (September 2018–May 2025) were retrospectively included and randomly divided into training and testing cohorts (7:3). Patients from the Third Affiliated Hospital of Xuzhou Medical University served as the external validation cohort. Least absolute shrinkage and selection operator (LASSO) regression was used to select predictors. Logistic regression (LR), random forest (RF), support vector machine (SVM), gradient boosting machine (GBM), and extreme gradient boosting (XGBoost) models were constructed. Model performance was assessed using receiver operating characteristic (ROC) curves, calibration plots, and decision curve analysis (DCA). SHapley additive explanations (SHAP) values were applied to interpret variable contributions, and an online prediction tool was developed.

**Results:**

A total of 1,454 patients were enrolled, of whom 731 developed adenoma recurrence within three years. LASSO identified ten predictors: infection of Helicobacter pylori (H. pylori), the number of adenomas, age, triglyceride level, body mass index (BMI), diarrhea, smoking history, family history, adenoma size, and fecal occult blood positivity. GBM achieved the highest mean AUC in repeated cross-validation (0.813) and maintained robust discrimination in the testing (AUC = 0.818) and external validation cohorts (AUC = 0.775), with good calibration and clinical utility. SHAP analysis identified H. pylori infection, adenoma number, and age as the leading contributors to model predictions.

**Conclusion:**

GBM demonstrated favorable discrimination, calibration, and clinical utility across validation cohorts, supporting its use for individualized risk stratification. The resulting online calculator may further facilitate risk assessment and inform follow-up strategies in clinical practice.

## 1 Introduction

Colorectal cancer (CRC) is one of the most prevalent malignant tumors of the alimentary tract worldwide. Based on data from the International Agency for Research on Cancer (IARC) in 2022, it ranks third in global incidence and second in mortality among all malignancies (1). Adenomatous polyps are the primary precancerous lesions of CRC, and approximately 85%-90% of CRC cases develop through the “adenoma-carcinoma” pathway (2, 3). With the widespread adoption of colonoscopy screening, the early detection and removal of adenomatous polyps have become a key strategy in preventing CRC.

Endoscopic mucosal resection (EMR), as one of the most commonly used polypectomy techniques in current clinical practice, not only effectively reduces the incidence of CRC but also significantly decreases the risk of mortality (4). However, postoperative recurrence remains a non-negligible adverse outcome in clinical practice. Studies have confirmed that patients still face a high risk of recurrence and potential malignant transformation after EMR, with recurrence rates ranging from 30% to 50% (5, 6). The recurrence risk is not only associated with adenoma characteristics such as size, morphology, and number (7, 8) but also closely linked to patient-specific and lifestyles factors (9). For instance, obesity (10), smoking (11), and family history (12) have all been demonstrated to be significantly correlated with an increased risk of the recurrence and progression of colorectal polyps.

Guidelines generally recommend a 3-year follow-up after adenoma resection (13, 14). Clinical evidence indicates that the risk of recurrence gradually increases 1–2 years after surgery and peaks around the third year after resection (15, 16). Shortening the follow-up interval to 1–2 years increases the frequency of examinations and the associated complication risks for low-risk individuals (17). Conversely, extending it to 5 years may miss the peak recurrence period and elevate the risk of progression to high-grade lesions (18, 19). Therefore, it is imperative to develop a predictive tool that can integrate individual factors to achieve risk stratification and provide a basis for determining follow-up intervals. However, current clinical practice primarily relies on guidelines rather than on a prediction model for recurrence risk based on individualized data.

Accordingly, this study retrospectively collected clinical data from patients with colorectal polyps to identify factors associated with adenoma recurrence within 3 years after EMR. Using machine learning approaches, we developed and validated an interpretable 3-year recurrence prediction model, with the aim of guiding the scheduling of follow-up intervals and improving the management of adenomatous polyps.

## 2 Materials and methods

### 2.1 Patient source and ethical statement

**Figure 1:**
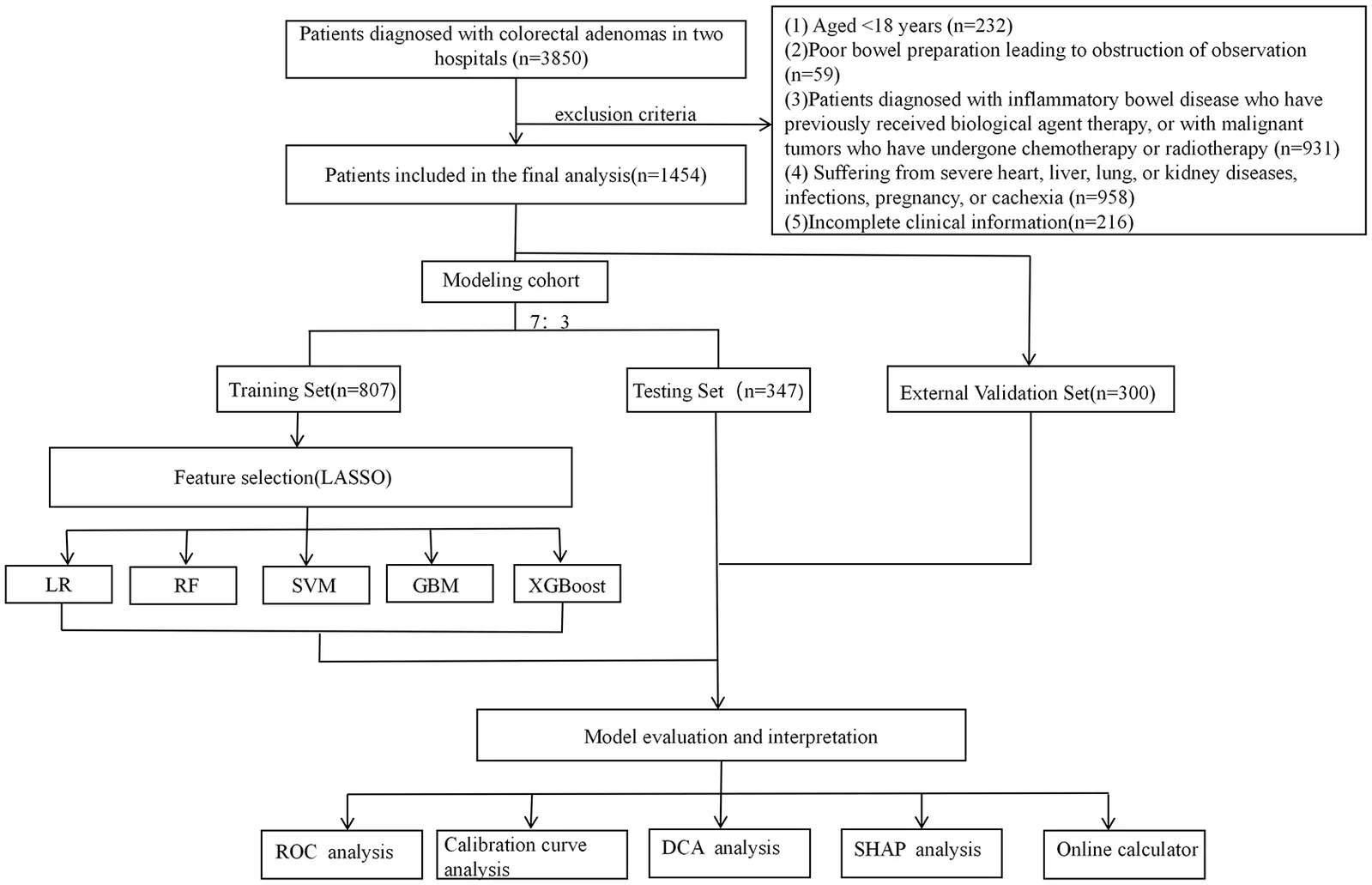
Overall workflow of the study. The workflow illustrates patient enrollment, cohort allocation, feature selection, model development and validation, model interpretation, and clinical application. LR, logistic regression; RF, random forest; SVM, support vector machine; GBM, gradient boosting machine; XGBoost, extreme gradient boosting; LASSO, least absolute shrinkage and selection operator; ROC, receiver operating characteristic; DCA, decision curve analysis; SHAP, SHapley additive explanations.

This study was a retrospective analysis (**Figure 1**). We reviewed the electronic medical records and follow-up data of patients treated at the Affiliated Hospital of Xuzhou Medical University between September 2018 and May 2025. Eligible patients underwent their initial EMR by May 2022, had histologically confirmed colorectal adenomas, and completed 3 years of follow-up. Recurrence was defined as the detection of a new adenoma at any surveillance colonoscopy within 3 years after EMR, regardless of previous negative findings; non-recurrence was defined as no adenoma detected during this period. Demographic, polyp-related, and laboratory data at the initial treatment were collected. The external validation cohort comprised patients from the Third Affiliated Hospital of Xuzhou Medical University who met the same eligibility criteria and had complete 3-year follow-up data available through August 2025. This study followed the Declaration of Helsinki and was approved by the Ethics Committee of the Affiliated Hospital of Xuzhou Medical University (Ethics Approval No. XYFY2025-KL260-01). Informed consent was waived due to the retrospective design.

### 2.2 Eligibility criteria

#### 2.2.1 Inclusion criteria

(1) Individuals aged ≥ 18 years who underwent their first gastrointestinal endoscopy; (2) patients with adequate intestinal preparation (Boston Bowel Preparation Scale (BBPS) score: ≥ 6) that did not compromise endoscopic observation or surgical operation by an endoscopist with at least three years of experience in endoscopic therapy; (3) patients initially diagnosed with adenomatous polyps postoperatively who received a 3-year colonoscopic follow-up after surgery; (4) those who underwent at least one surveillance colonoscopy at our hospital during the 3-year follow-up, with an interval of >1 year between examinations for those who had multiple colonoscopies; (5) patients with complete clinical materials, such as medical charts, endoscopic findings, laboratory data, and pathology reports.

#### 2.2.2 Exclusion criteria

(1) Patients who failed to receive a full colonoscopy because of poor bowel preparation; (2) patients diagnosed with inflammatory bowel disease or malignant tumors, or individuals who had received biological therapy, chemotherapy, or radiotherapy; (3) patients having severe cardiac, hepatic, pulmonary, or renal diseases, infections, pregnancy, or cachexia.

### 2.3 Predictors and definitions

(1) Demographics: sex, age, and body mass index (BMI).

(2) Clinical symptoms and history: diarrhea, hypertension, diabetes, smoking, drinking, family history of CRC, history of use of non-steroidal anti-inflammatory drug (NSAID), history of gallbladder disease, history of peptic ulcer, and concomitant gastric polyps.

(3) Laboratory parameters: infection of helicobacter pylori (H. pylori) (defined as a positive result on the urea breath test or serological antibodies test, and no history of prior eradication therapy), total cholesterol, triglycerides, and fecal occult blood (OB).

(4) Characteristics of polyps: Locations of adenomas (20) included proximal colons, distal colons, or whole colons. The proximal colons included ileocecal junctions, ascending colons, hepatic flexures, and transverse colons. The distal colons included splenic flexures, descending colons, sigmoid colons, and recta. Whole colon involvement referred to multiple polyps concurrently presenting in both the proximal and distal segments.

(5) Adenoma number and size: Multiple adenomas were defined as the presence of two or more lesions. Size was determined based on the diameter of the largest polyp.

(6) Risk stratification (21): Non-advanced adenomas were defined as tubular adenomas less than 10 mm in diameter with no high-grade intraepithelial neoplasia. Advanced adenomas included those with a diameter of 10 mm or above, tubulovillous/villous adenomas, or those with high-grade dysplasia.

### 2.4 Outcomes

Recurrence and non-recurrence of an adenomatous polyp: Recurrence was defined as the detection of a new adenomatous polyp within 3 years after the initial EMR. In contrast, non-recurrence was defined as no endoscopically detected new adenomatous polyp within 3 years after EMR for the initial adenomatous polyp.

### 2.5 Model development and validation

#### 2.5.1 Dataset partitioning and preprocessing

This study included patients who underwent EMR at the Affiliated Hospital of Xuzhou Medical University between September 2018 and May 2025. These patients constituted the development cohort and were randomly divided into training and testing cohorts at a 7:3 ratio. An external validation cohort comprised patients from the Third Affiliated Hospital of Xuzhou Medical University from September 2018 to August 2025, and it was used to assess the generalizability of the model. Missing values were imputed using the random forest (RF) method implemented in the mice package in R (22). The development cohort was imputed before the training–testing split, with the external-validation cohort processed separately. Five imputations (m = 5) were performed with a random seed of 123 without predictive mean matching; all missing rates were <1.3%.

#### 2.5.2 Feature selection

Feature variables were selected within the training cohort using least absolute shrinkage and selection operator (LASSO) regression. Binary categorical variables were coded as 0/1, while multilevel categorical variables were represented using predefined numerical codes; one-hot encoding was not employed (**Supplementary Table 1**). By applying L1 regularization, LASSO shrank less informative coefficients toward zero and retained the most relevant predictors for 3-year adenoma recurrence (23).

#### 2.5.3 Model construction and evaluation

Based on the selected predictors, five models were developed: logistic regression (LR), RF, support vector machine (SVM), gradient boosting machine (GBM), and extreme gradient boosting (XGBoost). Models were trained and tuned using stratified 10-fold cross-validation repeated five times with identical resampling partitions, and hyperparameters were selected by grid search based on the mean cross-validated area under the curve (AUC). Discrimination was evaluated using AUC, sensitivity, specificity, accuracy, positive predictive value (PPV), negative predictive value (NPV), and F1 score, and AUCs were compared using the DeLong test. Calibration was assessed using calibration curves, calibration intercept, calibration slope, and Brier score, and clinical utility was evaluated using decision curve analysis. For each model, the optimal probability threshold was determined by maximizing the Youden index based on pooled out-of-fold predictions from the training phase. This threshold was then fixed for evaluation in the testing and external validation cohorts. Threshold-dependent metrics were calculated using these fixed thresholds.

#### 2.5.4 SHAP-based model interpretability analysis

The SHapley additive explanations (SHAP) method was used to quantify the contribution of each feature to model predictions (24), thereby enhancing the interpretability of models. Additionally, SHAP plots were generated to characterize feature contributions at both the global and individual levels.

### 2.6 Statistical analysis

All statistical analyses, model development, and data visualization were implemented using R software (version 4.4.1). The baseline characteristics of patients were compared using the tableone package. Normally distributed continuous variables were expressed as mean ± standard deviation (Mean ± SD) and were compared using one-way analysis of variance (one-way ANOVA). In contrast, non-normally distributed continuous variables were expressed as median and interquartile range [Median (IQR)] and were compared using Kruskal-Wallis H test. Categorical variables were expressed as frequency and percentage [n (%)] and were compared using χ² test or Fisher’s exact test. P < 0.05 was deemed statistically significant.

## 3 Results

### 3.1 Baseline characteristics

Based on the inclusion and exclusion criteria, 1,454 patients were retrospectively enrolled in this study. Among them, 1,154 were from the Affiliated Hospital of Xuzhou Medical University, with 807 allocated to the training cohort and 347 to the testing cohort. An additional 300 patients from the Third Affiliated Hospital of Xuzhou Medical University constituted the external validation cohort. This study contained a small number of missing values at the missing rates of 0.09% for OB, 0.17% for BMI, 0.52% for history of gastric polyps, 0.52% for history of gallbladder disease, and 1.21% for history of peptic ulcer disease. The imputation of missing data is presented in **Supplementary Figure 1**. Baseline characteristics were generally comparable across the three cohorts, although significant differences were observed in gallbladder disease, NSAIDs use, hypertension, BMI, and polyp number (all *P* < 0.05; **Table 1**), indicating some degree of real-world inter-cohort heterogeneity.

**Table 1:** Baseline demographic and clinical characteristics of patients in the three cohorts, presented as n (%).

Variables	Overall (n=1454)	Training set (n = 807)	Testing set (n = 347)	External validation set (n = 300)	p
Gender					
Female	501 (34.5)	262 (32.5)	132 (38.0)	107 (35.7)	0.167
Male	953 (65.5)	545 (67.5)	215 (62.0)	193 (64.3)	
Family history					
No	1143 (78.6)	617 (76.5)	279 (80.4)	247 (82.3)	0.068
Yes	311 (21.4)	190 (23.5)	68 (19.6)	53 (17.7)	
Smoking history					
No	1066 (73.3)	577 (71.5)	258 (74.4)	231 (77.0)	0.163
Yes	388 (26.7)	230 (28.5)	89 (25.6)	69 (23.0)	
Alcohol consumption history					
No	1273 (87.6)	704 (87.2)	305 (87.9)	264 (88.0)	0.92
Yes	181 (12.4)	103 (12.8)	42 (12.1)	36 (12.0)	
Diabetes					
No	1203 (82.7)	672 (83.3)	285 (82.1)	246 (82.0)	0.834
Yes	251 (17.3)	135 (16.7)	62 (17.9)	54 (18.0)	
Diarrhea					
No	1064 (73.2)	577 (71.5)	257 (74.1)	230 (76.7)	0.206
Yes	390 (26.8)	230 (28.5)	90 (25.9)	70 (23.3)	
Pathological risk stratification					
non-advanced adenoma	1404 (96.6)	782 (96.9)	337 (97.1)	285 (95.0)	0.246
advanced adenoma	50 (3.4)	25 (3.1)	10 (2.9)	15 (5.0)	
Gallbladder disease					
No	1224 (84.2)	664 (82.3)	295 (85.0)	265 (88.3)	0.044
Yes	230 (15.8)	143 (17.7)	52 (15.0)	35 (11.7)	
H. pylori					
No	1002 (68.9)	539 (66.8)	246 (70.9)	217 (72.3)	0.137
Yes	452 (31.1)	268 (33.2)	101 (29.1)	83 (27.7)	
NSAIDs medication history					
No	1280 (88.0)	710 (88.0)	293 (84.4)	277 (92.3)	0.009
Yes	174 (12.0)	97 (12.0)	54 (15.6)	23 (7.7)	
Hypertension					
No	1120 (77.0)	597 (74.0)	270 (77.8)	253 (84.3)	0.001
Yes	334 (23.0)	210 (26.0)	77 (22.2)	47 (15.7)	
Age (mean (SD))	58.42 (10.60)	58.01 (10.41)	59.28 (10.28)	58.52 (11.41)	0.174
BMI (median [IQR])	23.10 [20.90, 25.30]	23.20 [21.10, 25.50]	23.10 [20.95, 25.30]	22.60 [20.70, 24.40]	0.013
Number (median [IQR])	2.00 [1.00, 3.00]	1.00 [1.00, 3.00]	2.00 [1.00, 3.00]	2.00 [1.00, 3.00]	0.011
Cholesterol (median [IQR])	4.72 [4.15, 5.22]	4.72 [4.12, 5.22]	4.72 [4.18, 5.23]	4.74 [4.17, 5.20]	0.876
Triglyceride (median [IQR])	1.21 [0.96, 1.65]	1.22 [0.96, 1.68]	1.18 [0.92, 1.60]	1.21 [0.98, 1.57]	0.36
OB					
No	1227 (84.4)	679 (84.1)	295 (85.0)	253 (84.3)	0.931
Yes	227 (15.6)	128 (15.9)	52 (15.0)	47 (15.7)	
Location					
Proximal colon	443 (30.5)	240 (29.7)	98 (28.2)	105 (35.0)	0.103
Distal colon	658 (45.3)	377 (46.7)	166 (47.8)	115 (38.3)	
Total colon	353 (24.3)	190 (23.5)	83 (23.9)	80 (26.7)	
Size					
< 0.5(cm)	1068 (73.5)	588 (72.9)	265 (76.4)	215 (71.7)	0.67
≥0.5 to ≤1.0 (cm)	325 (22.4)	186 (23.0)	68 (19.6)	71 (23.7)	
> 1(cm)	61 (4.2)	33 (4.1)	14 (4.0)	14 (4.7)	
Complicated with gastric polyps					
No	1289 (88.7)	707 (87.6)	306 (88.2)	276 (92.0)	0.117
Yes	165 (11.3)	100 (12.4)	41 (11.8)	24 (8.0)	
Peptic ulcer					
No	1286 (88.4)	702 (87.0)	310 (89.3)	274 (91.3)	0.111
Yes	168 (11.6)	105 (13.0)	37 (10.7)	26 (8.7)	

### 3.2 Predictor selection

**Figure 2:**
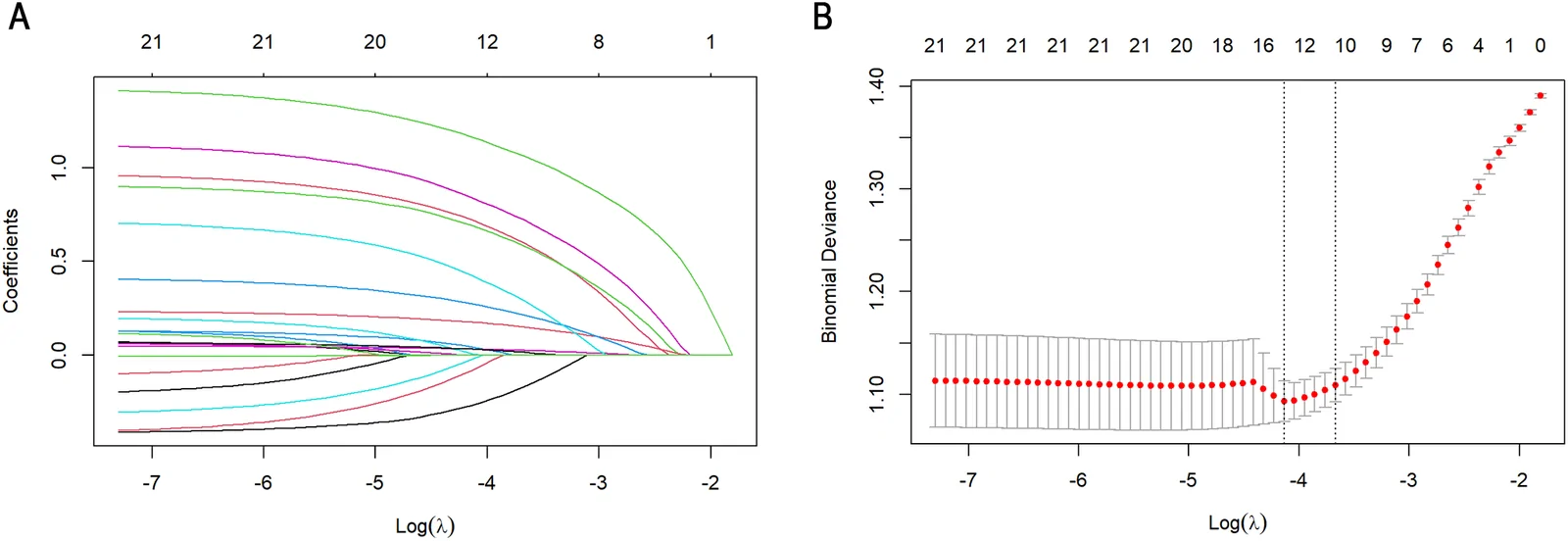
Feature selection using LASSO regression. **(A)** LASSO coefficient profiles of candidate predictors across different penalty parameters (λ). **(B)** Ten-fold cross-validation curve showing binomial deviance across λ values, with the optimal λ used to select the final predictors for model development.

In the training cohort, LASSO regression ultimately selected 10 feature variables (**Figures 2A, B**), namely infection of H. pylori, the number of adenomas, age, triglycerides, BMI, diarrhea, smoking, family history, adenoma size, and positive OB.

### 3.3 Model development and validation

Stratified ten-fold cross-validation repeated five times was performed in the 807-patient training cohort. All five models showed comparable discrimination, with mean cross-validated AUCs ranging from 0.802 to 0.813. GBM achieved the highest mean AUC (0.813; SD, 0.036; empirical 95% interval, 0.751–0.883), followed by XGBoost (0.809; SD, 0.036; 0.741–0.870), logistic regression (0.806; SD, 0.036; 0.739–0.866), SVM (0.803; SD, 0.038; 0.735–0.860), and random forest (0.802; SD, 0.039; 0.727–0.859) (**Supplementary Table 2**). Based on the optimal hyperparameters identified through cross-validation, the final models were refitted using the entire training cohort and evaluated in the held-out testing and external validation cohorts.

**Figure 3:**
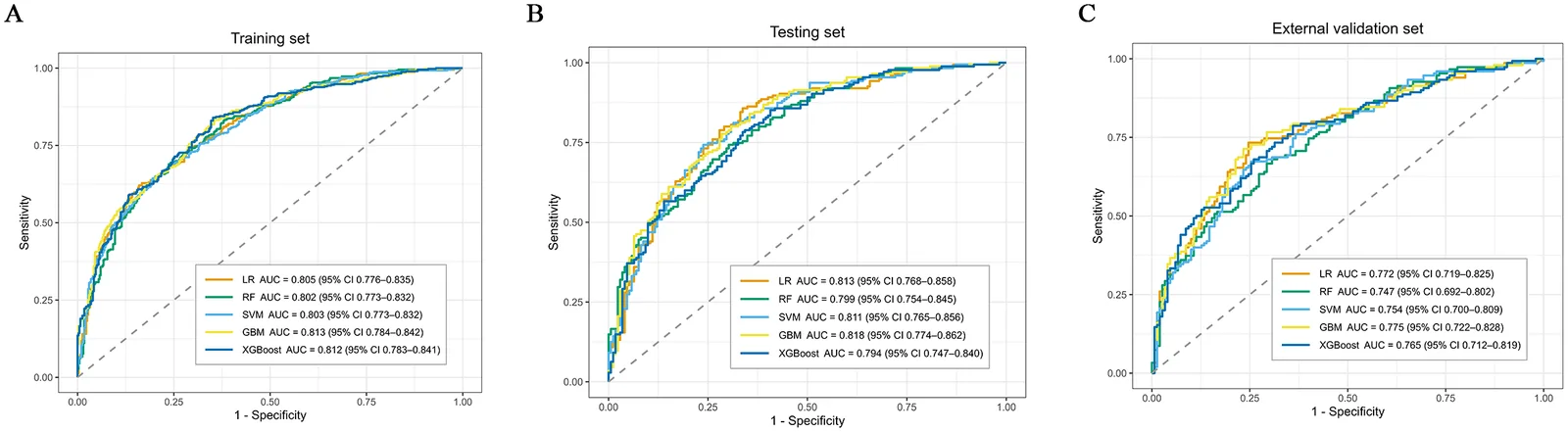
ROC curves of the five machine learning models across multiple datasets. **(A)** Aggregated out-of-fold ROC curves in the training cohort. **(B)** Testing cohort. **(C)** External validation cohort. The ROC curves are color-coded by model (LR, orange; RF, green; SVM, light blue; GBM, yellow; XGBoost, dark blue).

Receiver operating characteristic (ROC) curves for the final models are shown in **Figures 3A–C**. In the testing cohort, GBM achieved the highest AUC (0.818; 95% CI, 0.774–0.862), followed by LR (0.813; 95% CI, 0.768–0.858), SVM (0.811; 95% CI, 0.765–0.856), RF (0.799; 95% CI, 0.754–0.845), and XGBoost (0.794; 95% CI, 0.747–0.840). Compared with GBM, the difference was statistically significant only for XGBoost (*P* = 0.002), whereas the differences for LR, RF, and SVM were not significant (all *P*>0.05). In the external-validation cohort, GBM again achieved the highest AUC (0.775; 95% CI, 0.722–0.828), followed by LR (0.772; 95% CI, 0.719–0.825), XGBoost (0.765; 95% CI, 0.712–0.819), SVM (0.754; 95% CI, 0.700–0.809), and RF (0.747; 95% CI, 0.692–0.802). None of the pairwise differences compared with GBM reached statistical significance (all *P*>0.05; **Supplementary Table 3**). Considering its consistently favorable discrimination across repeated cross-validation, testing, and external validation, GBM was selected as the primary model for subsequent evaluation and interpretation. Model-specific cutoffs and the corresponding sensitivity, specificity, PPV, NPV, accuracy, and F1 scores are presented in **Table 2**.

**Table 2:** Performance comparison of the five machine learning models in the training, testing, and external validation sets.

Dataset	Model	AUC	Cutoff	SEN	SPE	PPV	NPV	ACC	F1
Training set (OOF predictions)	LR	0.805	0.591	0.621	0.840	0.797	0.686	0.730	0.698
	RF	0.802	0.502	0.690	0.763	0.747	0.708	0.726	0.717
	SVM	0.803	0.576	0.643	0.805	0.770	0.690	0.724	0.701
	GBM	0.813	0.392	0.830	0.646	0.704	0.790	0.739	0.762
	XGBoost	0.812	0.385	0.840	0.648	0.707	0.800	0.745	0.768
Testing set	LR	0.813	0.591	0.571	0.855	0.800	0.662	0.712	0.667
	RF	0.799	0.502	0.651	0.767	0.740	0.684	0.709	0.693
	SVM	0.811	0.576	0.611	0.837	0.793	0.679	0.723	0.690
	GBM	0.818	0.392	0.794	0.692	0.724	0.768	0.744	0.757
	XGBoost	0.794	0.385	0.783	0.657	0.699	0.748	0.720	0.739
External set	LR	0.772	0.591	0.507	0.867	0.792	0.637	0.687	0.618
	RF	0.747	0.502	0.580	0.747	0.696	0.640	0.663	0.633
	SVM	0.754	0.576	0.547	0.820	0.752	0.644	0.683	0.633
	GBM	0.775	0.392	0.767	0.693	0.714	0.748	0.730	0.740
	XGBoost	0.765	0.385	0.793	0.620	0.676	0.750	0.707	0.730

AUC, area under the receiver operating characteristic curve; ACC, accuracy; F1, F1 score; NPV, negative predictive value; OOF, out-of-fold; PPV, positive predictive value; SEN, sensitivity; SPE, specificity.

### 3.4 Model evaluation

**Figure 4:**
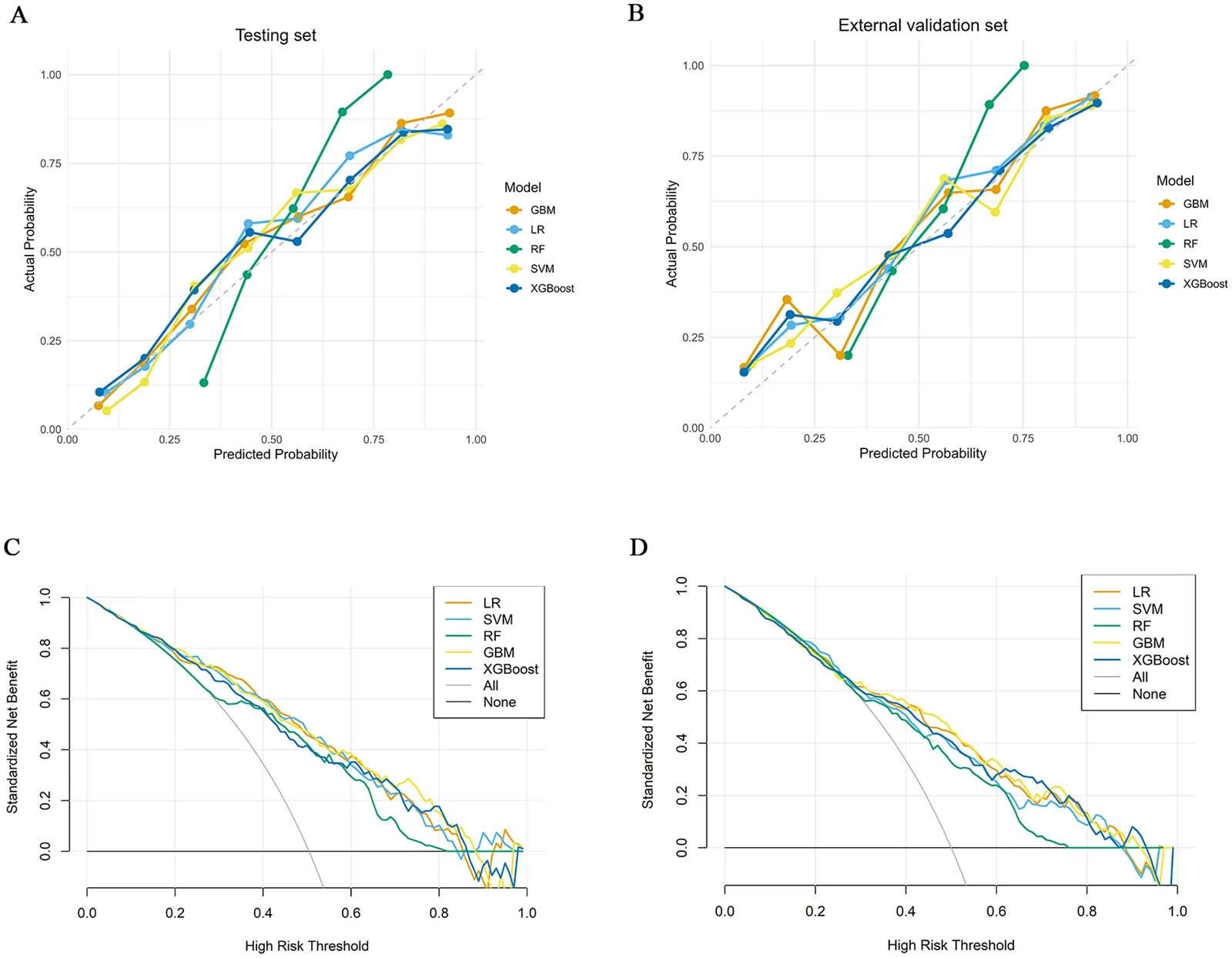
Calibration and decision curve analysis of the models. **(A)** Calibration curves of the testing set. **(B)** Calibration curves of the external validation set. **(C)** Decision curve analysis of the testing set. **(D)** Decision curve analysis of the external validation set. Calibration curves show agreement between predicted and observed outcomes, while decision curves illustrate the net benefit across different threshold probabilities.

Calibration curves demonstrated generally good agreement between predicted and observed outcomes in both the testing and external validation cohorts (**Figures 4A, B**). For GBM, the calibration intercept, slope, and Brier score were 0.116, 0.936, and 0.176 in the testing cohort and 0.227, 0.812, and 0.194 in the external validation cohort, respectively (**Supplementary Table 4**). Decision curve analysis demonstrated a positive net benefit across a range of threshold probabilities in both validation cohorts (**Figures 4C, D**); at the prespecified locked threshold of 0.392, GBM achieved a net benefit of 0.284 in the external validation cohort, compared with 0.177 for the treat-all strategy and 0 for the treat-none strategy. The GBM threshold derived from pooled out-of-fold training predictions using the Youden index was 0.392 and was fixed for subsequent validation. At this threshold, sensitivity, specificity, PPV, and NPV were 0.794, 0.692, 0.724, and 0.768 in the testing cohort and 0.767, 0.693, 0.714, and 0.748 in the external validation cohort, respectively (**Table 2**).

### 3.5 Model interpretation

**Figure 5:**
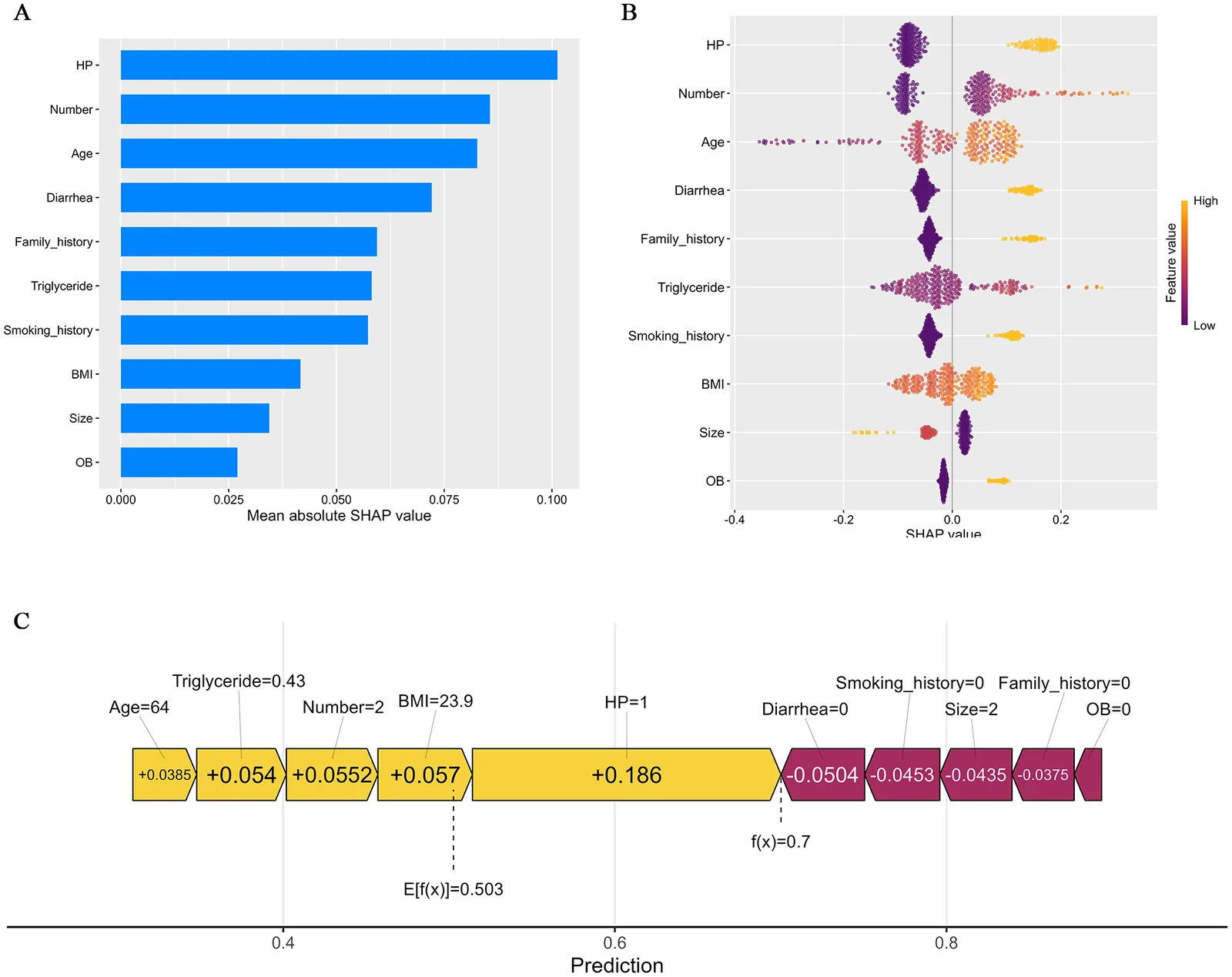
SHAP interpretation of the GBM model. **(A)** SHAP summary bar plot ranking features by mean absolute SHAP value. **(B)** SHAP beeswarm plot showing feature contributions for all samples; yellow indicates high feature values and purple indicates low feature values. **(C)** SHAP force plot illustrating how individual features contribute to the prediction of a single sample. This example is for illustrative purposes only and is not intended to guide clinical decision-making.

To enhance model interpretability, we employed SHAP to evaluate the contribution of each feature to model predictions. In the SHAP bar graph (**Figure 5A**), the features were sorted by their mean absolute SHAP values, with infection of H. pylori, the number of adenomas, and age identified as the top three contributors. The beeswarm plot (**Figure 5B**) further illustrated the direction and magnitude of individual feature contributions. H. pylori infection, greater adenoma number, diarrhea, family history, and smoking history were generally associated with higher predicted recurrence risk. Additionally, SHAP force plots were utilized to analyze the prediction mechanisms for individual samples. As shown in **Figure 5C**, an individual SHAP force plot was used as an illustrative example to demonstrate how patient-specific features contributed to a single GBM prediction. In this case, H. pylori infection was the strongest positive contributor, followed by BMI, adenoma number, triglyceride level, and age, whereas the absence of diarrhea, smoking history, and family history contributed negatively.

Overall, the directions of the main SHAP contributions were generally consistent with established clinical evidence. The single-patient force plot was presented for illustrative purposes only and was not intended for clinical decision-making.

### 3.6 Development of an online web calculator

**Figure 6:**
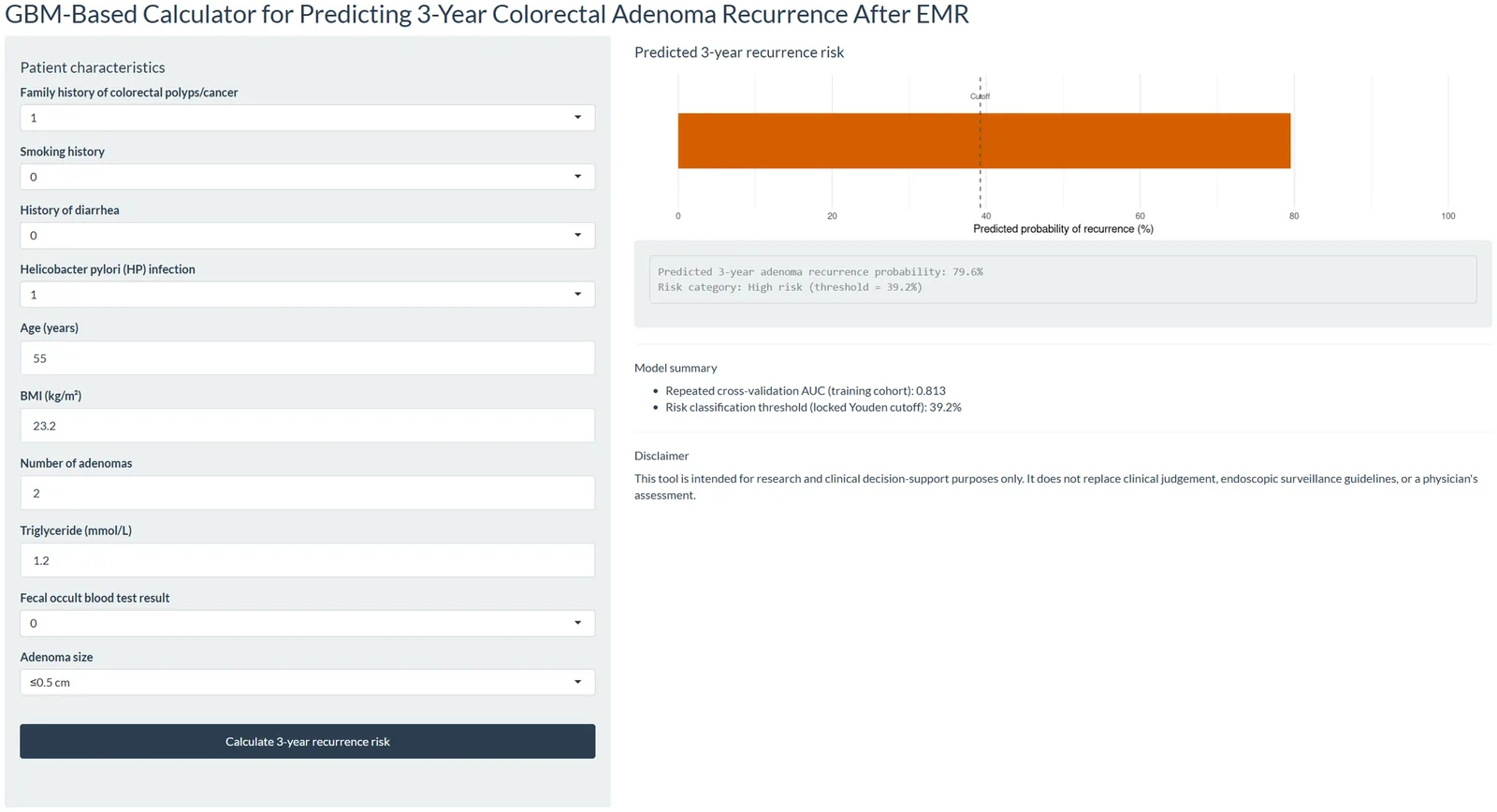
Online risk calculator for predicting colorectal adenoma recurrence within 3 years after endoscopic mucosal resection (EMR).

To facilitate clinical interpretation, we developed an online GBM-based calculator (https://plotsite.shinyapps.io/2026-08/) to estimate the 3-year recurrence probability after EMR. In the illustrative case shown in **Figure 6**, the predicted 3-year recurrence probability was 79.6%, exceeding the prespecified threshold of 39.2% and therefore corresponding to the higher-risk category. Although the GBM model was retrospectively evaluated in an independent external validation cohort, the calculator itself has not been prospectively evaluated for clinical impact. Therefore, it should be considered a risk-stratification tool rather than a stand-alone basis for surveillance decisions.

## 4 Discussion

In this multicenter retrospective study, we identified ten predictors associated with the recurrence of adenomatous colorectal polyps using LASSO regression and subsequently developed five machine learning-based prediction models. Among these models, GBM demonstrated the most favorable overall validation performance, with the highest AUC, a low Brier score, and acceptable calibration in the external-validation cohort. SHAP analysis was then applied to quantify the contribution of each predictor, and an online calculator was ultimately constructed to facilitate clinical implementation.

In recent years, the growing clinical demand for precise prediction has led to the increasingly widespread application of machine learning in medical research due to its exceptional capabilities of feature learning and prediction. Traditional regression models (such as nomograms) are still widely used owing to their advantages in visualization. For instance, a nomogram model developed by Li et al. based on the neutrophil-to-lymphocyte ratio (NLR) and triglyceride-glucose (TyG) index achieves an AUC of 0.762 (95% CI: 0.718-0.805) (25) for predicting adenoma recurrence. However, adenoma recurrence is influenced by multiple factors, including demographic characteristics, laboratory indicators, polyp features, and lifestyles (26). Significant nonlinear relationships and interaction effects exist among these variables (27). Therefore, it is difficult for traditional linear models to fully capture this complexity. Machine learning models can identify nonlinear relationships and implicit interactions among high-dimensional features, better aligning with the clinical reality of “multiple factors jointly driving disease progression” (28, 29). For example, Shi et al. have applied machine learning to predict short-term recurrence of adenomatous polyps, achieving promising results in multicenter data (30). Based on this, the current study employed machine learning to develop a prediction model for adenoma recurrence within three years after EMR. In the present study, GBM showed the most consistently favorable overall validation performance. It achieved the numerically highest AUC in both the testing (0.818) and external validation cohorts (0.775), along with favorable calibration and low prediction error. Despite some inter-cohort differences in baseline characteristics, GBM maintained favorable performance in the independent external cohort, suggesting a degree of robustness to inter-cohort heterogeneity. DeLong tests showed that GBM significantly outperformed XGBoost in the testing cohort (*P* = 0.002), whereas its differences from LR, RF, and SVM were not statistically significant. In the external validation cohort, no significant differences were observed between GBM and the other models. Notably, simpler models such as LR also demonstrated comparable discrimination, with AUCs of 0.813 in the testing cohort and 0.772 in the external validation cohort. The modest reduction in external validation performance may reflect inter-center heterogeneity in patient characteristics and clinical practice, highlighting the need for further prospective multicenter validation and local recalibration where necessary.

Among the predictors selected for model development, H. pylori infection emerged as an important factor associated with adenoma recurrence. H. pylori induces chronic inflammation by releasing urease and vacuolating cytotoxin A (VacA), elevating inflammatory cytokines such as tumor necrosis factor-alpha (TNF-α) and interleukin-6 (IL-6) (31, 32). These factors can influence the colorectal mucosal barrier and epithelial proliferation via the bloodstream, thereby increasing the risk of development and recurrence of adenomas (33). Similarly, research by Hu et al. has demonstrated that H. pylori eradication can reduce the recurrence rate of adenomas by 28% (HR = 0.72, 95% CI: 0.58-0.89) (34), further supporting its potential intervention value. The number of adenomas is also a key predictive factor. Patients with multiple adenomas (≥2) have a significantly higher risk of recurrence (35), which aligns with the “field effect” theory (36). Specifically, even after visible lesions are resected, the microenvironment conducive to adenoma formation may persist. Furthermore, multiple lesions increase the risk of incomplete resection, thereby further promoting recurrence (37, 38). Larger polyps are also associated with higher risks, as their complex structures and greater operative difficulties may lead to residual mucosa or persistent local inflammation (39). Additionally, older age is associated with an increased risk of adenoma recurrence (40). With advancing age, the repair capacity of intestinal mucosa declines, telomeres in epithelial cells shorten, and deoxyribonucleic acid (DNA) damage accumulates, leading to a corresponding rise in the risk of adenoma recurrence (41). This study found that elevated triglycerides and obesity (increased BMI) were also positively correlated. This aligns with the mechanism by which metabolic syndrome promotes intestinal tumorigenesis through insulin resistance and oxidative stress (42, 43). Meanwhile, diarrhea, as a manifestation of intestinal dysfunction, suggests dysbacteriosis, indirectly contributing to recurrence (44). Evidence has revealed that proteobacteria and fusobacteria are elevated in the fecal samples of individuals with adenoma, while bifidobacterium, faecalibacterium, bacteroides, and romboutsia are reduced (45), further reflecting impaired intestinal immune homeostasis (46). Smoking can increase adenoma recurrence by inducing DNA damage in colorectal epithelial cells through carcinogens and affecting immune regulation (47). A family history of CRC reflects genetic susceptibility. A study by Fossi et al. (48) has indicated that individuals with a positive result of family history have a substantially elevated risk of recurrence (OR = 2.23, 95% CI = 1.04-4.79). OB can serve as an early warning indicator for recurrence. In agreement with research by Botteri et al. (49), a positive result is associated with a greater risk of advanced adenomas, implicitly indicating the association between OB positivity and adenomas.

In practical applications, machine learning models are frequently regarded as “black boxes” due to their limited interpretability, which may hinder clinical adoption (50). To enhance model interpretability, we applied the game theory-based SHAP method to provide both global and individual-level explanations. SHAP quantifies the contribution of each feature to model predictions without implying causal effects. Our SHAP analysis revealed that H. pylori infection, adenomas number, age, triglycerides, BMI, diarrhea, smoking, family history, adenoma size, and positive OB were key features. Their directional effects align with previous evidence and clinical experience. For instance, infection of H. pylori, multiple adenomas, obesity, and advanced age have been confirmed to be associated with an elevated risk of recurrence (51), whereas normal BMI, no smoking, and no family history suggest a lower risk. Furthermore, SHAP can uncover nonlinear interactions among features, such as the combined effects of age and metabolic indicators (triglycerides and BMI), further enriching the interpretative depth of the model. Nevertheless, these model-specific SHAP findings do not imply causal relationships and warrant further independent validation. Based on these insights, the model extends beyond risk prediction by providing greater transparency into the factors underlying individual predictions. To facilitate clinical application, we further developed an online GBM-based calculator to estimate individualized 3-year recurrence risk after EMR, potentially supporting risk stratification and personalized follow-up planning. However, given the potential selection bias, the calculator should primarily be considered a risk-stratification tool and may not be directly generalizable to populations with different baseline risks or surveillance practices.

This study has several limitations. First, its retrospective design may have introduced selection bias. Patients with identified recurrence tended to have more complete 3-year follow-up, which may partly explain the relatively high recurrence rate (~50%). Although complete follow-up ensured reliable outcome assessment, the observed recurrence rate and absolute risk estimates may not be directly generalizable to populations with different surveillance patterns or baseline risks. Second, dynamic lifestyle factors, such as postoperative diet (52) and exercise levels (53) were not captured and may influence adenoma recurrence. Third, although missingness was low (<1.3%), multiple imputation by chained equations (MICE) was performed before the training-test split. Therefore, the possibility of limited information leakage and potential effects of missing-data handling cannot be completely excluded. Moreover, future prospective studies with standardized follow-up, external validation, and recalibration where necessary, are warranted to further assess the model’s generalizability and clinical applicability.

## 5 Conclusion

This study developed and externally validated machine learning models for predicting 3-year adenoma recurrence after EMR. GBM showed favorable overall performance and was further implemented as an online risk-stratification tool, although its discrimination was comparable to that of several alternative models. This tool may facilitate individualized follow-up planning. However, further prospective multicenter validation and local calibration are warranted before routine clinical use.

## Data Availability

The original contributions presented in the study are included in the article/**Supplementary Material**. Further inquiries can be directed to the corresponding authors.

## References

[1] Bray F Laversanne M Sung H Ferlay J Siegel RL Soerjomataram I . Global cancer statistics 2022: GLOBOCAN estimates of incidence and mortality worldwide for 36 cancers in 185 countries. CA Cancer J Clin. (2024) 74:229–63. doi: 10.3322/caac.21834 38572751

[2] van Toledo D IJspeert JEG Bossuyt PMM Bleijenberg AGC van Leerdam ME van der Vlugt M . Serrated polyp detection and risk of interval post-colonoscopy colorectal cancer: a population-based study. Lancet Gastroenterol Hepatol. (2022) 7:747–54. doi: 10.1016/s2468-1253(22)00090-5 35550250

[3] Sung JJY Chiu HM Lieberman D Kuipers EJ Rutter MD Macrae F . Third Asia-Pacific consensus recommendations on colorectal cancer screening and postpolypectomy surveillance. Gut. (2022) 71:2152–66. doi: 10.1136/gutjnl-2022-327377 36002247

[4] Ferlitsch M Hassan C Bisschops R Bhandari P Dinis-Ribeiro M Risio M . Colorectal polypectomy and endoscopic mucosal resection: European Society of Gastrointestinal Endoscopy (ESGE) Guideline - Update 2024. Endoscopy. (2024) 56:516–45. doi: 10.1055/a-2304-3219 38670139

[5] Cai S Shi H Fan M Zhang Q Lin R . Risk of adenoma recurrence after polypectomy in patients younger than 50 years vs. 50 years old and over with diminutive or small adenomas. Front Oncol. (2022) 12:823263. doi: 10.3389/fonc.2022.823263 36387065 PMC9641228

[6] Kemper G Turan AS Schoon EJ Schrauwen RWM Epping LSM Gerges C . Endoscopic techniques to reduce recurrence rates after colorectal EMR: systematic review and meta-analysis. Surg Endosc. (2021) 35:5422–9. doi: 10.1007/s00464-021-08574-z 34076765 PMC8437853

[7] Valle L Monahan KJ . Genetic predisposition to gastrointestinal polyposis: syndromes, tumour features, genetic testing, and clinical management. Lancet Gastroenterol Hepatol. (2024) 9:68–82. doi: 10.1016/s2468-1253(23)00240-6 37931640

[8] Zessner-Spitzenberg J Waldmann E Rockenbauer LM Demschik A Penz D Trauner M . Polyp size is associated with colorectal cancer death across histologic polyp subtypes: a retrospective study of a screening colonoscopy registry. Endoscopy. (2024) 56:820–7. doi: 10.1055/a-2339-0146 38936414

[9] Chi Z Lin Y Huang J Lv MY Chen J Chen X . Risk factors for recurrence of colorectal conventional adenoma and serrated polyp. Gastroenterol Rep (Oxf). (2022) 10:goab038. doi: 10.1093/gastro/goab038 35382162 PMC8972988

[10] Salimian S Habibi M Sehat M Hajian A . Obesity and incidence of colorectal polyps: a case-controlled study. Ann Med Surg (Lond). (2023) 85:306–10. doi: 10.1097/ms9.0000000000000234 36845814 PMC9949871

[11] Fagunwa IO Loughrey MB Coleman HG . Alcohol, smoking and the risk of premalignant and Malignant colorectal neoplasms. Best Pract Res Clin Gastroenterol. (2017) 31:561–8. doi: 10.1016/j.bpg.2017.09.012 29195676

[12] Hu Y Kharazmi E Liang Q Brenner H Sundquist J Sundquist K . Risk of colorectal cancer by family history of both colorectal carcinomas and colorectal polyps: a nationwide cohort study. Cancer Commun (Lond). (2025) 45:1407–16. doi: 10.1002/cac2.70059 40898381 PMC12629855

[13] Tanaka S Saitoh Y Matsuda T Igarashi M Matsumoto T Iwao Y . Evidence-based clinical practice guidelines for management of colorectal polyps. J Gastroenterol. (2021) 56:323–35. doi: 10.1007/s00535-014-1021-4 33710392 PMC8005396

[14] Hassan C Quintero E Dumonceau JM Regula J Brandão C Chaussade S . Post-polypectomy colonoscopy surveillance: European Society of Gastrointestinal Endoscopy (ESGE) Guideline. Endoscopy. (2013) 45:842–51. doi: 10.1055/s-0033-1344548 24030244

[15] Polychronidis G He MM Vithayathil M Knudsen MD Wang K Song M . Risk of colorectal neoplasia after removal of conventional adenomas and serrated polyps: a comprehensive evaluation of risk factors and surveillance use. Gut. (2024) 73:1675–83. doi: 10.1136/gutjnl-2023-331729 38839270

[16] Cross AJ Robbins EC Pack K Stenson I Rutter MD Veitch AM . Post-polypectomy surveillance interval and advanced neoplasia detection rates: a multicenter, retrospective cohort study. Endoscopy. (2022) 54:948–58. doi: 10.1055/a-1795-4673 35405762 PMC9500009

[17] Paszat LF Sutradhar R Luo J Rabeneck L Tinmouth J . Perforation and post-polypectomy bleeding complicating colonoscopy in a population-based screening program. Endosc Int Open. (2021) 9:E637–e645. doi: 10.1055/a-1381-7149 33880399 PMC8050559

[18] van Heijningen EM Lansdorp-Vogelaar I Steyerberg EW Goede SL Dekker E Lesterhuis W . Adherence to surveillance guidelines after removal of colorectal adenomas: a large, community-based study. Gut. (2015) 64:1584–92. doi: 10.1136/gutjnl-2013-306453 25586057 PMC4602240

[19] Vanbiervliet G Moss A Arvanitakis M Arnelo U Beyna T Busch O . Endoscopic management of superficial nonampullary duodenal tumors: European Society of Gastrointestinal Endoscopy (ESGE) Guideline. Endoscopy. (2021) 53:522–34. doi: 10.1055/a-1442-2395 33822331

[20] Laiyemo AO Doubeni C Sanderson AK Pinsky PF Doria-Rose VP Bresalier R . Likelihood of missed and recurrent adenomas in the proximal versus the distal colon. Gastrointest Endosc. (2011) 74:253–61. doi: 10.1016/j.gie.2011.02.023 21549375 PMC3148340

[21] Gupta S Lieberman D Anderson JC Burke CA Dominitz JA Kaltenbach T . Recommendations for follow-up after colonoscopy and polypectomy: A consensus update by the US multi-society task force on colorectal cancer. Gastroenterology. (2020) 158:1131–1153.e1135. doi: 10.14309/ajg.0000000000000544 32044092 PMC7672705

[22] Laqueur HS Shev AB Kagawa RMC . SuperMICE: an ensemble machine learning approach to multiple imputation by chained equations. Am J Epidemiol. (2022) 191:516–25. doi: 10.1093/aje/kwab271 34788362

[23] Wang Q Qiao W Zhang H Liu B Li J Zang C . Nomogram established on account of Lasso-Cox regression for predicting recurrence in patients with early-stage hepatocellular carcinoma. Front Immunol. (2022) 13:1019638. doi: 10.3389/fimmu.2022.1019638 36505501 PMC9726717

[24] Song YX Yang XD Luo YG Ouyang CL Yu Y Ma YL . Comparison of logistic regression and machine learning methods for predicting postoperative delirium in elderly patients: A retrospective study. CNS Neurosci Ther. (2023) 29:158–67. doi: 10.2139/ssrn.4123191 PMC9804041 36217732

[25] Li Z Zhao D Zhu C . Predicting colorectal adenoma recurrence: the role of systemic inflammatory markers and insulin resistance. Scand J Gastroenterol. (2025) 60:300–6. doi: 10.1080/00365521.2025.2469801 40009759

[26] Anderson JC Calderwood AH Christensen BC Robinson CM Amos CI Butterly L . Smoking and other risk factors in individuals with synchronous conventional high-risk adenomas and clinically significant serrated polyps. Am J Gastroenterol. (2018) 113:1828–35. doi: 10.1038/s41395-018-0393-0 30385834 PMC6768665

[27] Xie Y Jin Y Liu Z Li J Tao Q Wu Y . Identification of diagnostic biomarkers for colorectal polyps based on noninvasive urinary metabolite screening and construction of a nomogram. Cancer Med. (2025) 14:e70762. doi: 10.1002/cam4.70762 40200572 PMC11978731

[28] Kagawa Y . Clinical implications of a machine learning model predicting colorectal polyp recurrence after endoscopic mucosal resection. World J Gastroenterol. (2025) 31:107197. doi: 10.3748/wjg.v31.i22.107197 40539196 PMC12175858

[29] Handelman GS Kok HK Chandra RV Razavi AH Lee MJ Asadi H . eDoctor: machine learning and the future of medicine. J Intern Med. (2018) 284:603–19. doi: 10.1111/joim.12822 30102808

[30] Shi YH Liu JL Cheng CC Li WL Sun H Zhou XL . Construction and validation of machine learning-based predictive model for colorectal polyp recurrence one year after endoscopic mucosal resection. World J Gastroenterol. (2025) 31:102387. doi: 10.3748/wjg.v31.i11.102387 40124266 PMC11924002

[31] Xin H Zhu C Zhu C Zhang X Chen D Wang Q . Beyond the stomach: the association between Helicobacter pylori and the spectrum of digestive cancers. Front Cell Infect Microbiol. (2025) 15:1633227. doi: 10.3389/fcimb.2025.1633227 40964055 PMC12436461

[32] Lu D Wang M Ke X Wang Q Wang J Li D . Association between H. pylori infection and colorectal polyps: A meta-analysis of observational studies. Front Med (Lausanne). (2021) 8:706036. doi: 10.3389/fmed.2021.706036 35118081 PMC8803908

[33] Choi DS Seo SI Shin WG Park CH . Risk for colorectal neoplasia in patients with helicobacter pylori infection: A systematic review and meta-analysis. Clin Transl Gastroenterol. (2020) 11:e00127. doi: 10.14309/ctg.0000000000000127 32032128 PMC7145030

[34] Hu KC Wu MS Chu CH Wang HY Lin SC Liu CC . Decreased colorectal adenoma risk after helicobacter pylori eradication: A retrospective cohort study. Clin Infect Dis. (2019) 68:2105–13. doi: 10.1093/cid/ciy591 30566695

[35] Jang J Park J Park SJ Park JJ Cheon JH Kim TI . Clinical characteristics and risk factors related to polyposis recurrence and advanced neoplasm development among patients with non-hereditary colorectal polyposis. Intest Res. (2023) 21:510–7. doi: 10.5217/ir.2022.00139 37248175 PMC10626020

[36] Zhou RW Harpaz N Itzkowitz SH Parsons RE . Molecular mechanisms in colitis-associated colorectal cancer. Oncogenesis. (2023) 12:48. doi: 10.1038/s41389-023-00492-0 37884500 PMC10603140

[37] Djinbachian R Iratni R Durand M Marques P von Renteln D . Rates of incomplete resection of 1- to 20-mm colorectal polyps: A systematic review and meta-analysis. Gastroenterology. (2020) 159:904–914.e912. doi: 10.1053/j.gastro.2020.05.018 32437747

[38] Nishida A Andoh A . The role of inflammation in cancer: mechanisms of tumor initiation, progression, and metastasis. Cells. (2025) 14(7):488. doi: 10.3390/cells14070488 40214442 PMC11987742

[39] Ikuta S Saito Y Takata S Nakatani Y Nagatomo I Shiba S . Variability in non-tumor areas of colorectal cancer patients as revealed by endoscopic intestinal step biopsies. Mol Cancer. (2024) 23:249. doi: 10.1186/s12943-024-02159-9 39511621 PMC11546198

[40] Saiken A Gu F . Lifestyle and lifestyle-related comorbidities independently associated with colorectal adenoma recurrence in elderly Chinese people. Clin Interv Aging. (2016) 11:801–5. doi: 10.2147/cia.s105472 27382263 PMC4918738

[41] Liu Y Ji Y Jiang R Fang C Shi G Cheng L . Reduced smooth muscle-fibroblasts transformation potentially decreases intestinal wound healing and colitis-associated cancer in ageing mice. Signal Transduct Target Ther. (2023) 8:294. doi: 10.1038/s41392-023-01554-w 37553378 PMC10409725

[42] La Vecchia M Clavenna MG Sculco M Sala G Marradi D Barberis E . Gut microbiota and metabolome signatures in obese and normal-weight patients with colorectal tumors. iScience. (2025) 28:112221. doi: 10.1016/j.isci.2025.112221 40230532 PMC11995084

[43] Liu B Wen P Gu X Weng R Liu S . Elevated serum triglyceride predicts recurrence of colorectal polyps in patients with advanced adenomas. Lipids Health Dis. (2020) 19:211. doi: 10.1186/s12944-020-01388-3 32967679 PMC7513493

[44] Yin LL Qi PQ Hu YF Fu XJ He RS Wang MM . Dysbiosis promotes recurrence of adenomatous polyps in the distal colorectum. World J Gastrointest Oncol. (2024) 16:3600–23. doi: 10.4251/wjgo.v16.i8.3600 39171160 PMC11334022

[45] Aprile F Bruno G Palma R Mascellino MT Panetta C Scalese G . Microbiota alterations in precancerous colon lesions: A systematic review. Cancers (Basel). (2021) 13(12):3061. doi: 10.3390/cancers13123061 34205378 PMC8234190

[46] Chen H Tong T Lu SY Ji L Xuan B Zhao G . Urea cycle activation triggered by host-microbiota maladaptation driving colorectal tumorigenesis. Cell Metab. (2023) 35:651–666.e657. doi: 10.1016/j.cmet.2023.03.003 36963394

[47] Sayed IM Chakraborty A Inouye K Dugan L Tocci S Advani I . E-cigarettes increase the risk of adenoma formation in murine colorectal cancer model. Arch Toxicol. (2025) 99:1223–36. doi: 10.1007/s00204-024-03932-x 39786590 PMC12860471

[48] Fossi S Bazzoli F Ricciardiello L Nicolini G Zagari RM Pozzato P . Incidence and recurrence rates of colorectal adenomas in first-degree asymptomatic relatives of patients with colon cancer. Am J Gastroenterol. (2001) 96:1601–4. doi: 10.1016/s0002-9270(01)02347-4 11374706

[49] Botteri E Crosta C Bagnardi V Tamayo D Sonzogni AM De Roberto G . Predictors of advanced colorectal neoplasia at initial and surveillance colonoscopy after positive screening immunochemical faecal occult blood test. Dig Liver Dis. (2016) 48:321–6. doi: 10.1016/j.dld.2015.11.020 26739617

[50] Petch J Di S Nelson W . Opening the black box: the promise and limitations of explainable machine learning in cardiology. Can J Cardiol. (2022) 38:204–13. doi: 10.1016/j.cjca.2021.09.004 34534619

[51] Kim J Nath K Schmidlin K Schaufelberger H Quattropani C Vannini S . Hierarchical contribution of individual lifestyle factors and their interactions on adenomatous and serrated polyp risk. J Gastroenterol. (2023) 58:856–67. doi: 10.1007/s00535-023-02004-8 37300599 PMC10423128

[52] Adnan D Khoshaba ER Abdel-Reheem MK Trinh JQ Cao Y Bishehsari F . Correction: Association of late eating with colorectal adenomas: a cross-sectional study. Eur J Nutr. (2025) 64:82. doi: 10.1007/s00394-024-03580-y 39904890

[53] Li J You L Xu Z Bai H Fei X Yang J . Healthy lifestyle and the risk of conventional adenomas and serrated polyps: Findings from a large colonoscopy screening population. Int J Cancer. (2022) 151:67–76. doi: 10.1002/ijc.33974 35191524

