# Supplementary material for: Development and validation of a machine learning model for predicting 3-year recurrence of adenomatous colorectal polyps after EMR: a multicenter study
Source: Front Oncol. 2026 Sep 9;16:1936137. doi: 10.3389/fonc.2026.1936137 (PMC13597317; doi:10.3389/fonc.2026.1936137)
Supplement: Supplementary file 1 [file SupplementaryFile1.docx]

**Supplementary Materials**

Supplementary Figure 1. Distribution of missing data across all variables.

Supplementary Table 1. Coding and definitions of candidate predictors and outcome

| **Role** | **Variable** | **Coding / value** | **Definition** |
| --- | --- | --- | --- |
| Outcome | Recurrence | 0 = No recurrence; 1 = Recurrence | Adenoma recurrence within 3 years after EMR |
| Predictor | Gender | 0 = Female; 1 = Male | Sex |
| Predictor | Family history | 0 = No; 1 = Yes | Family history |
| Predictor | Smoking history | 0 = No; 1 = Yes | History of smoking |
| Predictor | Alcohol consumption history | 0 = No; 1 = Yes | History of alcohol consumption |
| Predictor | Diabetes | 0 = No; 1 = Yes | History of diabetes |
| Predictor | Diarrhea | 0 = No; 1 = Yes | History/presence of diarrhea |
| Predictor | Pathological type | 0 = Non-advanced adenoma; 1 = Advanced adenoma | Adenoma pathological classification |
| Predictor | Gallbladder disease | 0 = No; 1 = Yes | History of gallbladder disease |
| Predictor | H. pylori | 0 = No; 1 = Yes | Helicobacter pylori status/history |
| Predictor | NSAIDs use | 0 = No; 1 = Yes | History of NSAIDs drug use |
| Predictor | Hypertension | 0 = No; 1 = Yes | History of hypertension |
| Predictor | OB | 0 = No; 1 = Yes | OB status as defined in the study dataset |
| Predictor | Lesion location | 1 = Right hemicolon; 2 = Left hemicolon; 3 = Entire colon | Anatomical distribution of adenomatous lesions |
| Predictor | Lesion size | 1 = <0.5 cm; 2 = ≥0.5 to ≤1.0 cm; 3 = >1.0 cm | Maximum lesion-size category |
| Predictor | Gastric polyps | 0 = No; 1 = Yes | History/presence of gastric polyps |
| Predictor | Peptic ulcer | 0 = No; 1 = Yes | History/presence of peptic ulcer |
| Predictor | Age | Original numerical value | Age |
| Predictor | BMI | Original numerical value (kg/m²) | Body mass index |
| Predictor | Adenoma number | Original numerical value | Number of adenomas |
| Predictor | Total cholesterol | Original numerical value | Serum total cholesterol |
| Predictor | Triglycerides | Original numerical value | Serum triglycerides |

Supplementary Table 2. Cross-validated performance of the five prediction models in the training set

| Model | Mean cross-validated AUC | SD | Empirical 95% interval |
| --- | --- | --- | --- |
| GBM | 0.813 | 0.036 | 0.751–0.883 |
| XGBoost | 0.809 | 0.036 | 0.741–0.870 |
| Logistic regression | 0.806 | 0.036 | 0.739–0.866 |
| SVM | 0.803 | 0.038 | 0.735–0.860 |
| Random forest | 0.802 | 0.039 | 0.727–0.859 |

Note: Performance was assessed using repeated stratified 10-fold cross-validation with five repeats. The mean AUC and empirical 95% interval were derived from the 50 held-out-fold AUCs, whereas Table 2 reports AUCs calculated from pooled out-of-fold predictions.

Abbreviations: AUC, area under the receiver operating characteristic curve; SD, standard deviation; GBM, gradient boosting machine; SVM, support vector machine; XGBoost, extreme gradient boosting.

Supplementary Table 3. Pairwise comparisons of model AUCs with GBM in the testing and external-validation datasets

| Dataset | Comparison | Comparator AUC | GBM AUC | DeLong P value |
| --- | --- | --- | --- | --- |
| Testing set | LR vs GBM | 0.813 | 0.818 | 0.533 |
| Testing set | RF vs GBM | 0.799 | 0.818 | 0.102 |
| Testing set | SVM vs GBM | 0.811 | 0.818 | 0.444 |
| Testing set | XGBoost vs GBM | 0.794 | 0.818 | 0.002 |
| External-validation set | LR vs GBM | 0.772 | 0.775 | 0.791 |
| External-validation set | RF vs GBM | 0.747 | 0.775 | 0.058 |
| External-validation set | SVM vs GBM | 0.754 | 0.775 | 0.140 |
| External-validation set | XGBoost vs GBM | 0.765 | 0.775 | 0.265 |

Note: Paired DeLong tests were performed using predictions from the hyperparameter-tuned models. Raw P values are reported for comparisons between GBM and each of the other models within each dataset, with *P* < 0.05 considered statistically significant.

Abbreviations: AUC, area under the receiver operating characteristic curve; GBM, gradient boosting machine; LR, logistic regression; RF, random forest; SVM, support vector machine; XGBoost, extreme gradient boosting.

Supplementary Table 4. Quantitative calibration performance of the hyperparameter-tuned models

| Dataset | Model | Calibration intercept | Calibration slope | Brier score |
| --- | --- | --- | --- | --- |
| Testing set | LR | 0.118 | 0.957 | 0.178 |
| Testing set | RF | 0.056 | 2.581 | 0.201 |
| Testing set | SVM | 0.071 | 0.989 | 0.179 |
| Testing set | GBM | 0.116 | 0.936 | 0.176 |
| Testing set | XGBoost | 0.130 | 0.827 | 0.187 |
| External-validation set | LR | 0.251 | 0.894 | 0.196 |
| External-validation set | RF | 0.074 | 2.162 | 0.215 |
| External-validation set | SVM | 0.181 | 0.844 | 0.203 |
| External-validation set | GBM | 0.227 | 0.812 | 0.194 |
| External-validation set | XGBoost | 0.175 | 0.733 | 0.199 |

Note: Ideal calibration corresponds to an intercept of 0 and a slope of 1. Lower Brier scores indicate better overall probabilistic accuracy.

Abbreviations: GBM, gradient boosting machine; LR, logistic regression; RF, random forest; SVM, support vector machine; XGBoost, extreme gradient boosting.
